# Supplementary material for: Artemisinin Stimulates Neuronal Cell Viability and Possess a Neuroprotective Effect In Vitro
Source: Molecules. 2025 Jan 6;30(1):198. doi: 10.3390/molecules30010198 (PMC11723108; doi:10.3390/molecules30010198)
Supplement: Supplementary file 1 [file molecules-30-00198-s001.zip › molecules-3375357-supplementary.pdf]

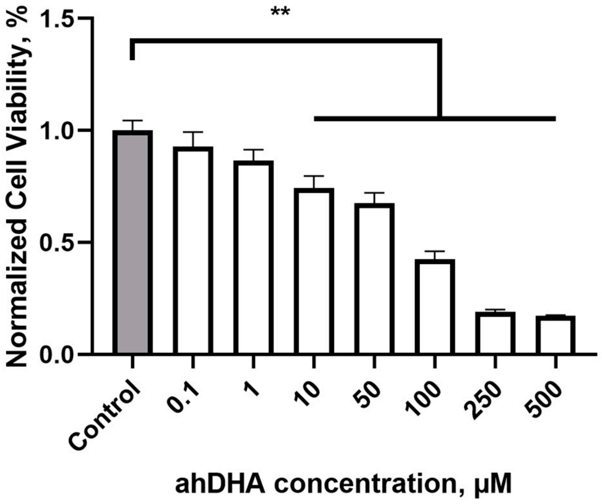

Figure S1. Effect of anhydrodihydroartemisinin (ahDHA) on SH-SY5Y cells' viability, proliferation, and ATP level. (**A**). Viability of SH-SY5Y cells 48 h after treatment, with anhydrodihydroartemisinin (ahDHA) across a range of micromolar concentrations measured using the MTS assay. Statistical analysis was performed using ANOVA, followed by Fisher's LSD test for multiple comparisons between groups; ** $p < 0.01$ compared to control.
